# Enamel interproximal reduction during treatment with clear aligners: digital planning versus OrthoCAD analysis

**DOI:** 10.1186/s12903-021-01487-2

**Published:** 2021-04-19

**Authors:** Giuseppina Laganà, Arianna Malara, Roberta Lione, Carlotta Danesi, Simonetta Meuli, Paola Cozza

**Affiliations:** Department of Systems Medicine, University of Rome ‘Tor Vergata’, Viale Oxford 81, 00133 Rome, Italy

**Keywords:** Dental crowding, Interproximal enamel reduction, Invisalign, ClinCheck

## Abstract

**Background:**

The aim of the study was to compare the amount of interproximal enamel reduction (IPR) provided on ClinCheck software with the amount of IPR carried out by the orthodontist during treatment with clear aligners.

**Methods:**

30 subjects (14 males, 16 females; mean age of 24.53 ± 13.41 years) randomly recruited from the Invisalign account of the Department of Orthodontics at the University of Rome “Tor Vergata” from November 2018 to October 2019, were collected according to the following inclusion criteria: mild to moderate dento-alveolar discrepancy (1.5–6.5 mm); Class I canine and molar relationship; full permanent dentition (excluding third molars); both arches treated only using Comprehensive Package by Invisalign system; treatment plan including IPR. Pre- (T0) and post-treatment (T1) digital models (.stl files), created from an iTero scan, were collected from all selected patients. The OrthoCAD digital software was used to measure tooth mesiodistal width in upper and lower arches before (T0) and at the end of treatment (T1) before any refinement. The widest mesio-distal diameter was measured for each tooth excluding molars by “Diagnostic” OrthoCAD tool. The total amount of IPR performed during treatment was obtained comparing the sum of mesio-distal widths of all measured teeth at T0 and T1. Significant T1–T0 differences were tested with dependent sample t-test (*P* < 0.05).

**Results:**

In the upper arch, IPR was digitally planned on average for 0.62 mm while in the lower arch was on average for 1.92 mm. As for the amount of enamel actually removed after IPR performing, it was on average 0.62 mm in the maxillary arch. In the mandibular arch, the mean of IPR carried out was 1.93 mm. The difference between planned IPR and performed IPR is described: this difference was on average 0.00 mm in the upper arch and 0.01 in the lower arch.

**Conclusions:**

The amount of enamel removed in vivo corresponded with the amount of IPR planned by the Orthodontist using ClinCheck software.

## Background

The Invisalign appliance was introduced for the first time to the public in the late 1990s by Align Technology as an innovative method to straightening teeth without braces [1]. This system uses impressions or intraoral scans which are converted through stereolithographic technology (.stl) into virtual models and then launched with the ClinCheck software: a three-dimensional modeling program that allows a virtual simulation of teeth movements. A series of aligners is then produced in order to gain the needed corrections [2].

Most of patients treated with Invisalign present dental crowding that is a key reason for people seeking orthodontic treatment. Enamel interproximal reduction (IPR), extractions, increase in arch perimeter with distalization, buccal arch expansion, or incisor protrusion are important therapy strategies to solve dental crowding [3]. The main target of an orthodontic treatment is to provide the best balance among occlusal relationships, dental and facial esthetics and long-term treatment stability. Achieving these goals could be difficult in most patients because of the excess of tooth structures that often can interfere with the correct alignment of the teeth in the dental arch [4]. In cases of mild to moderate dental crowding, IPR represents a good therapy solution. It is a clinical procedure that allows to gain space in order to align teeth through the reduction, the anatomic recontouring and the protection of interproximal enamel surfaces of permanent teeth [5]. This technique may help achievement of treatment goals while preserving the integrity of the dental and periodontal tissues [6]. In overall terms, a major contribution of IPR is that the extent of expansion in the labial direction can be reduced, thus reducing the risk of bone dehiscence [7]. In additional, enlarged proximal contacts stabilize the treatment result [8]. IPR also provides to the elimination of “black triangles” allowing an improvement in the aesthetic appearance [7]. Creating the proper apposition areas for the gingiva also reduces or prevents retrusion of the interdental papillae [9]. In Invisalign treatment IPR is pre-planned during ClinCheck development: the clinician can decide the amount of IPR to be performed and the area and stage where it is needed [5]. To prevent residue excessive space, persisting misaligned teeth, or inter-arch discrepancies, it is important to quantify the amount of enamel that can be removed [10]. Moreover, the amount of IPR actually done has to be as the expected and programmed one in order to obtain the planned movements [11].

This high accuracy is essential to reach treatment objectives particularly during 3D digitally treatment plans [10].

Despite the available literature relating Invisalign technology, IPR reliability has been analyzed less deeply and the results still remains unclear. Although many studies have focused on the surface irregularities that could remain after grinding and polishing [12, 13], only a few papers presenting a quantitative evaluation of stripped enamel have been identified [7, 14]. Johner et al. aimed to investigate the predictability of the expected amount of IPR using three different methods on premolars. The study showed large variations in the amount of enamel removed, in fact, in most cases, actual stripping was on average less than the expected amount of enamel reduction and the different stripping technique was not significant [14]. Recently De Felice et al. investigated the fidelity of the IPR performed by manually system, during clear aligners therapy, and showed that the amount of enamel removed in vivo did not correspond to the expected amount of IPR [11].

In this regard, the aim of the present study was to evaluate, in terms of enamel reduction, the accuracy and the correspondence of the IPR planned by ClinCheck software and the IPR carried out by the orthodontist by mechanical oscillating systems. The null hypothesis was that there is not a difference between the expected and the actual amounts of enamel reduction.

## Methods

This study followed the principles laid down by the World Medical Assembly in the Declaration of Helsinki 2008 on medical protocols and ethics and received positive response by the Ethic Committee at the University of Rome Tor Vergata (protocol number: 141/19). For the present retrospective study 30 subjects (14 males, 16 females; mean age of 24.53 ± 13.41 years) randomly recruited from the Invisalign account of the Department of Orthodontics at the University of Rome “Tor Vergata” from November 2018 to October 2019, were collected according to the following inclusion criteria:

- mild to moderate dento-alveolar discrepancy (1.5–6.5 mm);
- Class I canine and molar relationship;
- full permanent dentition (excluding third molars);
- both arches treated only using Comprehensive Package by Invisalign system;
- treatment plan including IPR.

Patients with dentofacial deformity or medical problems, poor compliance with aligners, extractions therapy, auxiliaries other than Invisalign attachments, Invisalign no Comprehensive Package therapy, multiple and/or advanced caries, impacted, missing, or supernumerary teeth, prosthetic restorations, were excluded. Written consent was obtained from all participating subjects.

For the treatment protocol, each subject was instructed to wear aligners for 22 h per day, except during meals and oral hygiene procedures and to replace aligners on average every 15 days. Every 6 stages the clinician checked the good aligner fitting and the position of the attachments. The mean number of aligners was 25 for the maxillary arch and 24 for the mandibular arch. Both arches averaged 8 attachments and less than 2.5 mm of IPR in the upper arch and less than 4.5 mm of IPR in the lower arch.

IPR was carried out by the same experienced operator (G.L.) at the programmed phase according to the virtual treatment staging under water-cooling. It was planned by ClinCheck software, from the mesial surface of the right second premolar to the mesial surface of the left second premolar in both arches, according to treatment needs of the single clinical case and then recorded in an Excel file. IPR was achieved using oscillating abrasive strip mounted in a contra-angle handpiece with rotation speed of 20,000 rpm as suggested by the manufacturer and the amount of space obtained was checked with metal gages. To amplify the remineralization capacity of the abraded proximal surfaces, a topical fluoride gel was applied on the reduced teeth for 5 min.

Pre- (T0) and post-treatment (T1) digital models (.stl files), created from an iTero scan, were collected from all selected patients.

### Data measurement

**Fig. 1:**
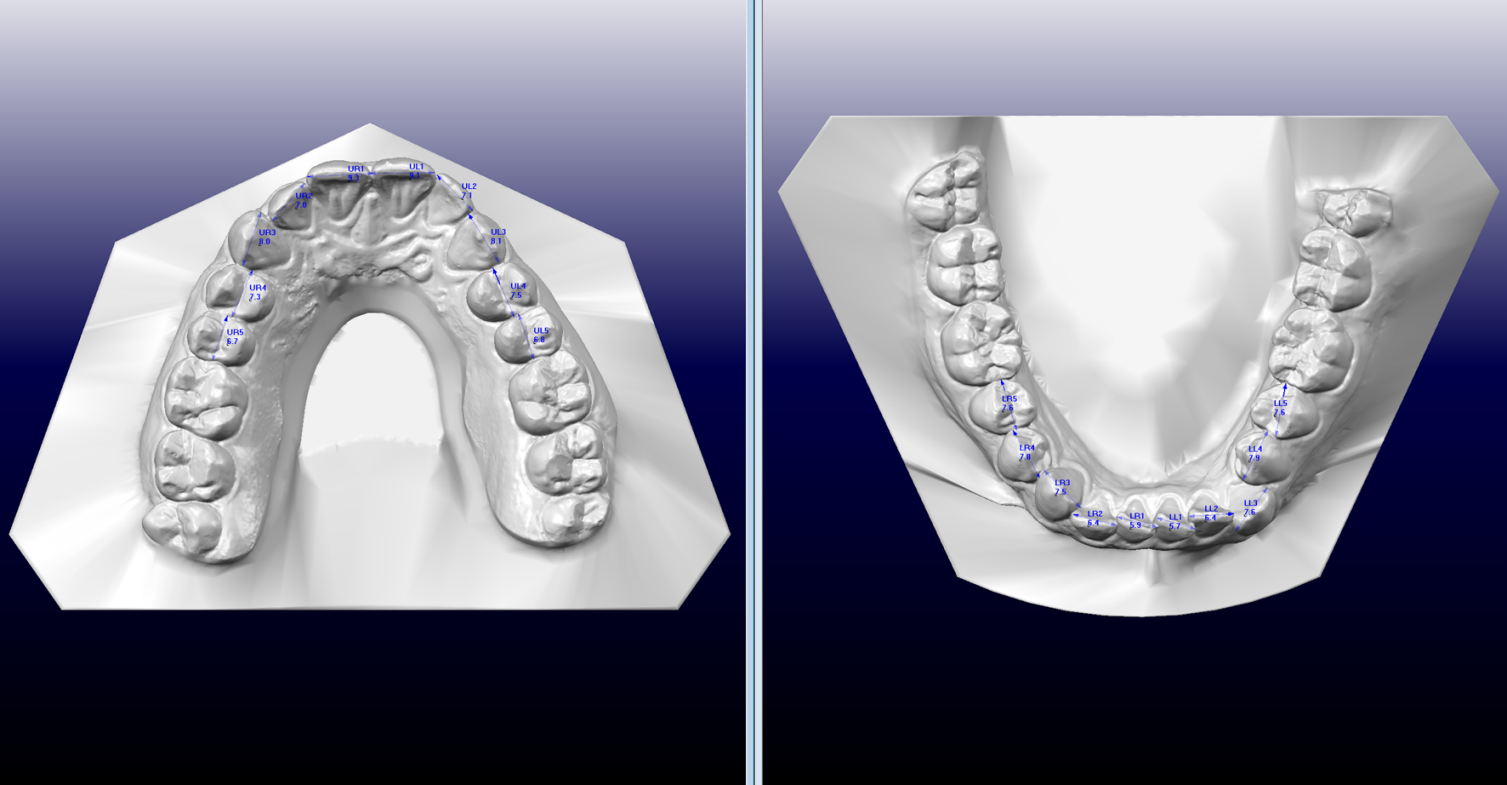
Mesio-distal diameters analysis by OrthoCAD software before the treatment (T0)

The pre-treatment and post-treatment .stl files were uploaded into OrthoCAD software (3Shape, Copenhagen, Denmark) version 5.9.0.36. The OrthoCAD digital software was used to measure tooth mesiodistal width in upper and lower arches before (T0) and at the end of treatment (T1) before any refinement (Fig. 1). The widest mesio-distal diameter was measured for each tooth excluding molars by “Diagnostic” OrthoCAD tool. The total amount of IPR performed during treatment was obtained comparing the sum of mesio-distal widths of all measured teeth at T0 and T1.

To determine the reliability of the method, all measurements were made by a single operator (A.M.) who was blinded to the entity of IPR planned and performed, and were checked by a second operator (S.M.). They were repeated 2 weeks after the first assessment. A paired *t*-test was used to compare the two measurements (systematic error). The magnitude of the random error was calculated by using the method of moment’s estimator [15].

### Statistical analysis

The power of the study for the independent sample *t*-test was calculated on the basis of the sample size of two groups and an effect size equal to 0.9 [16]. The power was 0.80 at an alpha level of 0.05 (SigmaStat 3.5, Systat Software, Point Richmond, California, USA).

In the presence of normally distributed data, a paired *t*-test was selected to compare the T1–T0 changes. The level of significance was set at 5%.

SPSS (Statistical Package for the Social Sciences), version 18.0 (IBM Corp, Chicago III) was the chosen software to analyze data.

## Results

No systematic error was found among the repeated digital measurements. The systematic error was reduced by precise definitions of points in the presence of a previously trained experienced examiner. The mean random error was 0.42 mm and within acceptable limits because the software allowed a more accurate view of the anatomic details. The mean time between the initial and the final scans was 11.87 months.

47 arches were collected for this investigation: in 17 patients interproximal enamel reduction was digitally planned and performed in both arches, in 2 subjects only in the maxillary arch while in 11 participants only in the mandibular arch. The mean of IPR digitally planned in the upper arch was 0.62 mm with values ranging from 0.20 to 2.20 mm, in the lower arch it was 1.92 mm with values ranging from 0.20 to 4.40 mm (Table 1). As for the amount of enamel actually removed after IPR performing, it was on average 0.62 mm in the maxillary arch. In the mandibular arch, the mean of IPR carried out was 1.93 mm (Table 1).

**Table 1:** Descriptive analysis of the study group

Study group n = 30	
*Age (years, months)*	
Mean	24.53
Min	11
Max	56
Gender	
M	14
F	16
*Upper arch*	
IPR planned (mm)	
Mean	0.62
Min	0.20
Max	2.20
IPR performed (mm)	
Mean	0.62
Min	0.20
Max	2.30
*Lower arch*	
IPR planned (mm)	
Mean	1.92
Min	0.20
Max	4.40
IPR performed (mm)	
Mean	1.93
Min	0.20
Max	4.50

In the Table 2 the difference between planned IPR and performed IPR is described: this difference was on average 0.00 mm in the upper arch and 0.01 in the lower arch.

**Table 2:** Descriptive statistics and statistical comparison between T0 and T1 differences by means of Student *t*-test for paired groups

	Mean of IPR digitally planned (T0) (mm)	Mean of IPR performed (T1) (mm)	T1–T0	95% CI	*p* values
Upper arch	0.62 ± 0.63	0.62 ± 0.63	0.00	− 0.16 to 0.16	NS
Lower arch	1.92 ± 1.93	1.93 ± 1.97	0.01	− 0.18 to 0.12	NS

*NS* not significant, *CI* confidence interval

No statistically significant differences were found in both upper and in lower arch.

## Discussion

The purpose of the present study was to verify, through a three-dimensional data set, the accuracy and the correspondence between the IPR provided by ClinCheck software and the IPR carried out by the orthodontist by mechanical oscillating systems. Therefore, careful pre-operative planning by using ClinCheck software is essential to determine where and when to perform IPR and its amount in order to achieve the expected results. IPR and staging are important items to manage on each and every patient. Proper IPR technique and staging of events such as new attachment placement can help ensure treatment proceeds smoothly and appropriately [17]. This study focused on the use of the oscillating abrasive strip mounted in a contra-angle handpiece to perform IPR. Oscillating strips are easy to use and provide precise enamel reduction, better visualization and access. They are available in several widths and are color-coded, offering control over specific point-of-contact reduction. IPR should be performed incrementally, proceeding through increasing widths to the desired amount rather than selecting the thickest width first. This modality is particularly useful in the anterior segments because of the ease of access. It is important not to use excessive pressure to avoid the risk of strip fracture and enamel surface protrusions [18].

IPR plays an important role in non-extraction treatment to obtain space to align teeth and/ or to achieve more long-term alignment stability [6]. So, knowledge of the predictability of this procedure is important to improve treatment outcomes for this technique. Nevertheless, there are very few studies in literature dealing with this purpose [7, 11, 14]. Surely the most important one, as well as recent and similar to ours is by De Felice et al. [11] whose main target was to compare the accuracy of the actual space obtained through interproximal enamel reduction to the amount of IPR planned through the digital setup during clear aligner treatment. Although the present study showed a concordance between planned and actually performed IPR, De Felice et al. demonstrated that the amount of enamel removed in vivo did not correspond with the amount of IPR planned and, indeed, in most cases the amount of IPR performed was lower. This difference can be attributed to two key factors. First, the IPR technique. De Felice et al. used the manual method with single-sided diamond-coated strips; in our study, instead, the stripping method consisted of a mechanical oscillating system. This might suggest a greater predictability of the amount of IPR and of the mechanical method that, anyway, was not the aim of the present study.

However, the greater efficacy and precision of IPR the mechanical method compared to the manual one is reported in literature by different papers [10, 13, 19]. Mechanical strips have been shown to be more efficient in reducing enamel than the manual system; in particular, the mechanical system reduced the inaccuracy of the manual one satisfying precision potentially down to 0.1 mm required by 3D treatment planes such as clear aligners [10]. The decrease in abrasive properties is significantly less considerable for mechanical IPR system and, as a matter of fact, according to the recent survey by Kaaouara et al. [20], the mechanical oscillating diamond strips produced a more regular surface, with light parallel lines and smaller grooves than manual abrasive strips, although a constant decrease has been observed for both systems [21].

Finally, another substantial difference can be found in the recruitment of the subjects in the study group. In our investigation, the patients were selected from a single Invisalign provider, while in the study by De Felice et al. the subjects were selected from ten different orthodontists. As it is recognized in literature, the amount of enamel removed by stripping is influenced by operator or technique factors including the pressure applied, the hardness, the size of the abrasive, the duration of IPR, and tooth-related aspects such as enamel hardness [22]. In order to guarantee standardization of the experimental IPR technique, in our study it was performed by a single clinician within a pre-established period, while closely following the manufacturers' instructions [23].

## Conclusions

The null hypothesis of the study is confirmed: the amount of enamel removed in vivo corresponds with the amount of IPR planned by the Orthodontist using ClinCheck software. This result could be due to the IPR method and to the high experience of the Orthodontist who performed it, as a matter of fact IPR is influenced by operator or technique factors [22].

## Data Availability

The datasets used and/or analysed during the current study are available from the corresponding author on reasonable request.
